# In the era of Bortezomib-based Induction, intensification of Melphalan-based conditioning with Bortezomib does not improve Survival Outcomes in newly diagnosed Multiple Myeloma: a study from the Chronic Malignancies Working Party of the EBMT

**DOI:** 10.1038/s41409-023-02160-8

**Published:** 2024-01-31

**Authors:** Meral Beksac, Diderik-Jan Eikema, Linda Koster, Cyrille Hulin, Xavier Poiré, Rose-Marie Hamladji, Tomasz Gromek, Ali Bazarbachi, Zubeyde Nur Ozkurt, Thomas Pabst, Tarek Ben Othman, Jürgen Finke, Olga Pirogova, Depei Wu, Amjad Hayat, Inken Hilgendorf, Eleni Tholouli, Liesbeth C. de Wreede, Stefan Schönland, Laurent Garderet, Joanna Drozd-Sokolowska, Kavita Raj, Patrick J. Hayden, Ibrahim Yakoub-Agha, Donal P. McLornan

**Affiliations:** 1 Istinye University Ankara Liv Hospital Hematology and Stem Cell Transplantation Unit, Ankara, 06880 Turkey; 2 EBMT Statistical Unit, Leiden, the Netherlands; 3 EBMT Leiden Study Unit, Leiden, the Netherlands; 4 CHU Bordeaux, Hopital Haut-Leveque, Pessac, France; 5 Cliniques Universitaires St. Luc, Brussels, Belgium; 6 Centre Pierre et Marie Curie, Alger, Algeria; 7 Samodzielny Publiczny, Lublin, Poland; 8 Bone Marrow Transplantation Program, American University of Beirut Medical Center, Beiruit, Lebanon; 9 Gazi University Faculty of Medicine, Ankara, Turkey; 10 University Hospital Bern, Bern, Switzerland; 11 Centre National de Greffe de Moelle, Tunis, Tunisia; 12 University of Freiburg, Freiburg, Germany; 13 RM Gorbacheva Research Institute, Pavlov University, St. Petersburg, Russian Federation; 14 First Affiliated Hospital of Soochow University, Suzhou, China; 15 The Blood and Tissue Establishment, Galway University Hospital, Galway, Ireland; 16 Universitaetsklinikum Jena, Jena, Germany; 17 Manchester Royal Infirmary, Manchester, UK; 18 Department of Biomedical Data Sciences, Leiden University Medical Center, Leiden, the Netherlands; 19 Medizinische Klinik u. Poliklinik V, University of Heidelberg, Heidelberg, Germany; 20 Hôpital Pitié Salpêtrière, Hematology department, Paris, France; 21 University Clinical Centre, Medical University of Warsaw, Warsaw, Poland; 22 Department of Stem Cell Transplantation, University College London Hospitals, London, UK; 23 Department of Haematology, Trinity College Dublin, St. James’s Hospital, Dublin, Ireland; 24 CHU de Lille, Univ Lille, INSERM U1286, Infinite, Lille, 59000 France

**Keywords:** Myeloma, Stem-cell therapies

## Abstract

Bortezomib (Vel)- Melphalan 200 mg/m2 (Mel200) (Vel-Mel) has been utilised to intensify conditioning in autologous hematopoietic stem cell transplantation (AHCT) for multiple myeloma (MM). This EBMT registry-based study compared Vel-Mel with Mel200 during upfront AHCT. Between 2010 and 2017, MM patients who received Vel-Mel (n = 292) conditioning were compared with 4,096 Mel200 patients in the same 58 centres. Pre-AHCT, compared to Mel200 patients, Vel-Mel patients had similar International Staging System (ISS) scores and cytogenetic risk profiles; a similar proportion had received bortezomib-based induction (85% and 87.3%, respectively) though they were younger with a better performance status. Vel-Mel patients were more likely to achieve CR post-induction (40.6% vs 20.3%, p < 0.001) and by day 100 of AHCT (CR/VGPR: 70.2 % vs. 57.2%, p < 0.001). There was no difference in 3-year PFS (49% vs 46%, p = 0.06) or early post-AHCT mortality. In multivariable analysis, Vel-Mel associated with inferior PFS (HR: 1.69 (1.27–2.25, p < 0.001) and OS (HR:1.46 (1.14–1.86,p = 0.002), similar to negative effects on PFS of advanced ISS (HR:1.56 (1.33–1.83, p < 0.001), high-risk cytogenetics (HR:1.43(1.18–1.74, p < 0.001) and poor post-induction response(<=PR)(HR: 1.43(1.25–1.62, p < 0.001) Overall, despite superior pre- and post-AHCT responses, there was no improvement in PFS or OS following Vel-Mel. This data supports the findings of the smaller prospective IFM study.

## Introduction

The deep responses achieved with potent modern induction regimens in patients with newly diagnosed multiple myeloma (NDMM) has led some to question the role and timing of Autologous Hematopoietic Stem Cell Transplantation (AHCT). Two recent studies comparing early versus late AHCT, have demonstrated that transplantation confers superior progression-free survival (PFS) in all patients and superior overall survival (OS) in high-risk patients [1, 2]. Attempts to improve the efficacy of AHCT and reduce relapse rates are ongoing. As regards the intensity of conditioning, Bensinger and colleagues compared Melphalan 200 mg/m2 (Mel200) versus Melphalan 280 mg/m2 (Mel280) combined with Amifostine as conditioning regimens in a randomised study [3]. Although the intensified regimen conferred superior response rates (Mel 200 vs. Mel280; near complete response (⩾nCR) 22% vs 39%, P = 0.03, ⩾ partial response (PR) 57% vs. 74%, P = 0.04), the PFS rates at 1- and 3-years were 83% and 46% for Mel200, and 78% and 54% for Mel280, respectively. A European Society for Blood and Marrow Transplantation (EBMT) study which compared outcomes following two different Melphalan doses (Mel 140 mg/m2 (Mel140) vs. Mel 200) found no significant difference in OS, PFS, the cumulative incidence of relapse, non-relapse mortality (NRM), hematopoietic recovery and second primary malignancy (SPM) rates between the two groups [4]. However, regarding disease status pre-AHCT, patients in PR or less appeared to benefit from the higher dose (adjusted hazard ratios (HR) for Mel200 versus Mel140: 0.5, 0.54, and 0.56). In contrast, OS was superior in the Mel140 cohort for those who were in a very good partial response (VGPR) or a CR (adjusted HR: 2.02). Other approaches to intensify melphalan-based containing regimens have included the addition of busulfan, bendamustine, bortezomib, lenalidomide, carfilzomib and, more recently, ixazomib [5–12]. Bortezomib has been the most widely used novel drug based on both in vitro and in vivo data showing synergistic activity [13, 14]. Early reports from the Intergroupe Francophone du Myélome (IFM) and a phase I/II study by Lonial et al. highlighted the safety of a Vel-Mel AHCT conditioning regimen [15, 16]. Since then, Jethava et al. and Nishhori et al. have additionally reported potentially positive benefits [17, 18]. A PETHEMA study followed a different approach and combined Bortezomib within Busulfan and Melphalan (BuMelVel) [19, 20]. More recently, however, the IFM group reported outcomes from a phase III randomized study comparing Vel-Mel (Vel (1.0 mg/m^2^ IV on days −6, –3, +1, and +4 and Mel200 on day –2) with Mel200 alone [21]. This prospective open label study reported that the Vel-Mel regimen conferred no advantage in terms of efficacy end points, PFS or OS. Given the lack of reported data on the real-world use of Vel-Mel conditioning in the upfront setting, this retrospective registry-based analysis aimed to compare outcomes of NDMM patients transplanted with Vel-Mel and Mel200.

## Methods

### Patient selection

This is a retrospective, registry-based study, approved by the Chronic Malignancies Working Party (CMWP) of the EBMT. All patients whose transplant data are reported to EBMT have provided informed consent for this information to be used in anonymized research projects. All adult NDMM patients who received a first AHCT between January 1^st^, 2010, and December 31^st^, 2017, and who received Vel-Mel conditioning were eligible for inclusion in the study. In the EBMT database 33711 patients treated with Melphalan 200 mg/msq (Mel200) were identified. The main comparison in the study was between Mel200 conditioning alone and Mel200 + Bortezomib (Vel-Mel), subsequently, patients transplanted in centres that treated patients with Mel200 and Vel-Mel were included (58 centres, n = 4388). It was noted that the total doses of Bortezomib administered were in accordance with those given in the IFM studies comparing Vel-Mel with Mel200. EBMT centres commit to obtain informed consent according to the local regulations applicable at the time of transplantation in order to report pseudonymised data to the EBMT.

### Statistics

All time-to-event analyses were conducted from the date of the first AHCT. The primary endpoints in the study were OS and PFS, and were estimated using the Kaplan-Meier product limit estimation method. Median follow-up was determined using the reverse Kaplan-Meier method. Three-year outcomes after the start of patient follow-up were of specific interest and any events occurring after three years from the start of patient follow-up were censored. Differences in subgroups were assessed by means of the Log-Rank test. The cumulative incidences of CR/VGPR and PR after AHCT-1 were analysed in a competing risks framework with competing event death due to any cause. Subgroup differences in cumulative incidences were assessed using Gray’s test. Multivariable (MVA) Cox regression was applied to investigate the impact of Vel-Mel vs. Mel200 on OS and PFS, adjusting for possible confounders. Identical covariate constellations were used for both models, and included covariates were as follows: conditioning at first AHCT (Vel-Mel versus Mel200), disease status prior to conditioning (VGPR, <=PR versus CR), patient age at AHCT (in decades), ISS (II, III versus I), Karnofsky score at AHCT (90–100 versus <90) and Cytogenetics by Fluorescence In Situ Hybridization (FISH) (high-risk (del(17p), t(4;14) or t(14;16)) versus standard risk (absence of del(17p), t(4;14) and t(14;16) abnormalities)). Missing values on adjustment factors other than disease status prior to conditioning were handled using the missing indicator method. Non-proportionality in the effect of conditioning was handled using time dependent coefficients, where appropriate. Continuous variables are presented in the text as median and interquartile ranges (IQR) and categorical variables as percentages within the group of patients with available data. Subgroup differences were evaluated by the χ2 test for categorical variables and t-tests for continuous data. All survival estimates and hazard ratios are reported with corresponding 95% confidence intervals in parentheses. All univariable analyses of outcomes are based on complete cases. Frequencies and percentages of missing values are presented where applicable. All p-values were two-sided and p < 0.05 was considered significant. Statistical analyses were performed in R version 3.6.0 (R Development Core Team, Vienna, Austria), using packages ‘survival’, ‘prodlim’ and ‘cmprsk’.

## Results

### Patients

A total of 4,388 patients from 58 EBMT-affiliated centres, 292 (6.7%) of whom had received Vel-Mel200 and 4,096 (93.3%) Mel200, were included in the study. Pre-transplantation characteristics are summarized in Table 1. The proportion of patients who received bortezomib-based induction regimens were similar (85% of Mel200 and 87.3% of Vel-Mel). Vel-Mel patients were younger (median age 56.8 vs. 59 years, p < 0.001) and more fit (Karnofsky score ≥90: 78.9% vs 67.2%, p < 0.001), at the time of AHCT-1. Fewer were of IgG isotype (66.7 % vs 72.7%, p < 0.001). Vel-Mel patients achieved higher post-induction response rates (CR: 40.6% vs. 20.3% and VGPR: 22.9% vs. 39.6% respectively, p < 0.001). FISH cytogenetic results were available for 1,589 patients. The frequency of high-risk cytogenetics was similar (22.3% in Vel-Mel and 19.6% in Mel200, p = 0.6). Although the proportion of del(17p) (6.6% in Mel200 vs. 5.3% in Vel-Mel, p = 0.8) and t(4;14) (9.6% in Mel200 vs.14.9% in Vel-Mel, p = 0.14) were similar, t(14;16) was more frequent in the Vel-Mel group (5.3 % vs. 1.1%, p = 0.004).

**Table 1:** Baseline characteristics whole cohort and stratified by donor type.

	Group	Missing	Total	Mel200	Vel-Mel	P
			N (%)	N (%)	N (%)	
Total			4388 (100%)	4096 (93.3%)	292 (6.7%)	
Age at AHCT-1	Median (IQR)		58.9 (53–63.5)	59 (53.3–63.5)	56.8 (50.7–62.8)	<0.001
Patient sex	Male		2536 (57.8%)	2366 (57.8%)	170 (58.2%)	0.928
	Female		1852 (42.2%)	1730 (42.2%)	122 (41.8%)	
Karnofsky score	<90	285 (6.5%)	1312 (32%)	1252 (32.8%)	60 (21.1%)	<0.001
	90–100		2791 (68%)	2566 (67.2%)	225 (78.9%)	
ISS	I	2235 (50.9%)	926 (43.0%)	857 (43.3%)	69 (39.7%)	0.504
	II		682 (31.7%)	627 (31.7%)	55 (31.6%)	
	III		545 (25.3%)	495 (25.0%)	50 (28.7%)	
Ig type	IgG	1303 (29.7%)	2229 (72.3%)	2089 (72.7%)	140 (66.7%)	<0.001
	IgA		751 (24.3%)	698 (24.3%)	53 (25.2%)	
	IgD/M/E		105 (3.4%)	88 (3.1%)	17 (8.1%)	
Cytogenetics	standard	2799 (63.8%)	1275 (80.2%)	1202 (80.4%)	73 (77.7%)	0.607
	high		314 (19.8%)	293 (19.6%)	21 (22.3%)	
AHCT-1 year	Median (IQR)		2014 (2012–2016)	2014 (2012–2016)	2014 (2012–2016)	0.033
Interval diagnosis AHCT-1 (months)	Median (IQR)	33 (0.8%)	6.3 (4.7–10.1)	6.2 (4.7–9.9)	7.7 (5.6–11.6)	0.871
Bortezomib induction	no	2943 (67.1%)	213 (14.7%)	193 (15.0%)	20 (12.7%)	0.507
	yes		1232 (85.3%)	1094 (85.0%)	138 (87.3%)	
Response at AHCT-1	CR/VGPR	53 (1.2%)	939 (21.7%)	822 (20.3%)	117 (40.6%)	<0.001
	<=PR		1668 (38.5%)	1602 (39.6%)	66 (22.9%)	
			1728 (39.9%)	1623 (40.1%)	105 (36.5%)	
Response at d100	CR/VGPR	511 (16.4%)	1533 (58.8%)	1375 (57.7%)	158 (70.2%)	<0.001
	<=PR		1074 (41.2%)	1007 (42.3%)	67 (29.8%)	

P-values were obtained using the χ2 test for categorical variables and t-tests for continuous data.

### Factors affecting transplant outcomes

The clinical outcomes of patients based on conditioning regimens are presented in Table 1. The Day +100 post-AHCT response rates (CR/VGPR) in patients alive and in follow-up at that time were superior in the Vel-Mel group (70.2% in Vel-Mel vs 57.7% in Mel200, p < 0.001). For patients who died before Day +100, death due to infection, organ damage/failure and toxicity was similar: 4/5 in the Vel-Mel group and 32/44 in the Mel200 group. Of those who proceeded to a second Mel200 or Vel-Mel AHCT within one year of the first, 39.8% (n = 317) were performed after Mel200 AHCT-1 (n = 796) and 76% (n = 19) after Vel-Mel AHCT-1 (n = 25). The median follow-up was 36.8 (35.2 - 38.4) months for all patients, 35.9 (34.1–37.5) months in Mel200 and 50.2 (45.4–55) months in Vel-Mel.

**Fig. 1:**
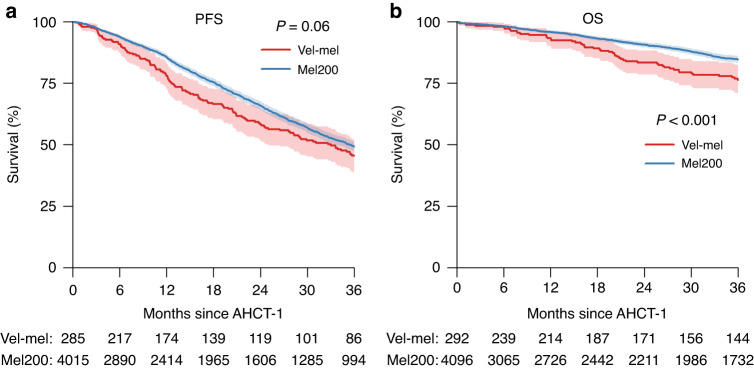
Kaplan Meier curves of PFS and OS stratified according to conditioning regimen of AHCT-1. Kaplan-Meier curves of (**a**) progression free survival (PFS) and (**b**) overall survival (OS) until 3 years after transplant, stratified by Melphalan (Mel) and Vel-Mel (Velcade + Melphalan) conditioning at first autologous hematopoeitic stem cell transplantation (AHCT-1). Corresponding log-rank p-values are indicated in the plots. Survival probabilities are represented as percentages, with the 95% confidence intervals indicated as shaded regions. The corresponding log-rank p-value is indicated in the plot. Below the time axis are the number of patients at risk at indicated timepoints, in each group.

As seen in Fig. 1, PFS estimates at three years post AHCT-1 were similar in the Vel-Mel and Mel200 cohorts (46% (39–52%) and 49% (47–51%) respectively, p = 0.06). OS at 3 years was significantly better in the Mel200 cohort when compared to Vel-Mel (85% (83–86%) vs 76% (71–82%), p < 0.001). As shown in Table 2 (Univariable analyses of OS and PFS after AHCT-1), lower ISS scores and the absence of high-risk cytogenetic abnormalities were associated with superior three-year PFS: ISS I/II/III (55% (51-59%)/ 48% (43–53%)/ 39% (34–44%), p < 0.001), cytogenetics (standard vs. High-risk, 50% (47–54%) vs. 39% (32–46%), p < 0.001). Similarly, better Karnofsky score at AHCT and better response at AHCT-1 were associated with significantly improved OS.

**Table 2:** Univariable analyses of overall survival (OS) and progression free survival (PFS) after first AHCT, overall and stratified by relevant risk factors.

	Group	PFS			OS		
		N	3 y (95% CI)	P	N	3 y (95% CI)	P
**Overall**		4300	49% (47–51%)		4388	84% (83–85%)	
**Conditioning at AHCT**	Vel-Mel	285	46% (39–52%)	0.06	292	76% (71–82%)	<0.001
	Mel200	4015	49% (47–51%)		4096	85% (83–86%)	
**Age at AHCT**	<50	704	49% (44–53%)	0.9	721	85% (81–88%)	0.4
	50–64	2929	49% (47–51%)		2989	84% (83–86%)	
	65+	667	49% (44–54%)		678	82% (78–86%)	
**ISS**	I	922	55% (51–59%)	<0.001	926	91% (88–93%)	<0.001
	II	672	48% (43–53%)		682	84% (80–87%)	
	III	536	39% (34–44%)		545	78% (73–82%)	
**Karnofsky at AHCT**	<90	1282	48% (45–52%)	0.2	1312	82% (79–84%)	0.006
	90–100	2743	49% (47–51%)		2791	85% (83–86%)	
**Cytogenetics**	standard	1264	50% (47–54%)	<0.001	1275	87% (85–89%)	<0.001
	high	311	39% (32–46%)		314	77% (71–83%)	
**Ig**	IgG	2194	50% (47–52%)	0.06	2229	85% (83–87%)	0.1
	IgA	743	45% (41–50%)		751	81% (77–85%)	
	IgD/M/E	104	54% (43–66%)		105	85% (76–93%)	
**Response at AHCT**	CR/VGPR	2564	54% (51–56%)	<0.001	2607	86% (84–87%)	0.002
	<=PR	1687	43% (40–46%)		1728	82% (80–84%)	
**Light chain**	kappa	2727	50% (48–53%)	0.02	2782	86% (84–88%)	<0.001
	lambda	1443	47% (43–50%)		1474	80% (77–82%)	
	Non-secretory	50	62% (46–78%)		51	85% (72–97%)	

Three-year Kaplan Meier estimates are given, with group differences evaluated by means of logrank tests. All estimates are reported with 95% confidence intervals in parentheses.

Table 3 shows the results of MVA which found Vel-Mel conditioning to be an adverse factor for PFS (HR: 1.69 (1.27–2.25), p < 0.001) within the first year and for OS (HR: 1.46 (1.14–1.86), p = 0.002). The effect on PFS is no longer observed after 1 year post-AHCT. In addition, advanced ISS III compared to I (HR: 1.56 (1.33–1.83), p < 0.001), high-risk cytogenetics (HR:1.43 (1.18–1.74, p < 0.001) and inferior post-induction responses (<=PR HR 1.43 (1.25–1.62), p < 0.001) also adversely affected PFS. ISS III compared to I and high-risk cytogenetics adversely affected OS (HR 2.26 (1.72–2.96), p < 0.001 and HR 1.78 (1.33–2.38), p < 0.001 respectively). Age was an additional prognostic factor for OS only: HR: 1.11 (1.01–1.22), p = 0.037, per decade increase.

**Table 3:** Multivariable analysis of progression free survival (PFS) and overall survival (OS) after first autologous transplant (AHCT).

Risk factor	PFS				OS		
	Group	Follow-up	HR (95% CI)	P	Group	HR (95% CI)	P
Conditioning	Mel200	=<1 year	1		Mel200		
	Vel-Mel	=<1 year	1.69 (1.27–2.25)	<0.001	Vel-Mel	1.46 (1.14–1.86)	0.002
	Mel200	>1 year					
	Vel-Mel	>1 year	0.88 (0.71–1.10)	0.3			
Response pre-AHCT	CR				CR		
	VGPR		1.15 (1.00–1.31)	0.05	VGPR	0.8 (0.65–0.98)	0.034
	<=PR		1.43 (1.25–1.62)	<0.001	<=PR	1.13 (0.94–1.37)	0.2
Karnofsky at AHCT	<90				<90		
	90–100		0.95 (0.86–1.05)	0.3	90–100	0.87 (0.75–1.02)	0.09
Age at AHCT (decades)			0.99 (0.94–1.06)	0.8		1.11 (1.01–1.22)	0.037
ISS	I				I		
	II		1.27 (1.08–1.49)	0.003	II	1.79 (1.36–2.35)	<0.001
	III		1.56 (1.33–1.83)	<0.001	III	2.26 (1.72–2.96)	<0.001
FISH	Standard				Standard		
	High		1.43 (1.18–1.74)	<0.001	High	1.78 (1.33–2.38)	<0.001

Patient age at transplant is in decades. The non-proportional effect of conditioning in PFS was modeled by a step-function of time. Separate hazard ratios were estimated in the follow-up periods 0–1 year and >1 year after AHCT. The effect of conditioning was proportional in OS. Effect estimates are given with 95% confidence intervals. Corresponding p-values are calculated using the Wald test.

**Fig. 2:**
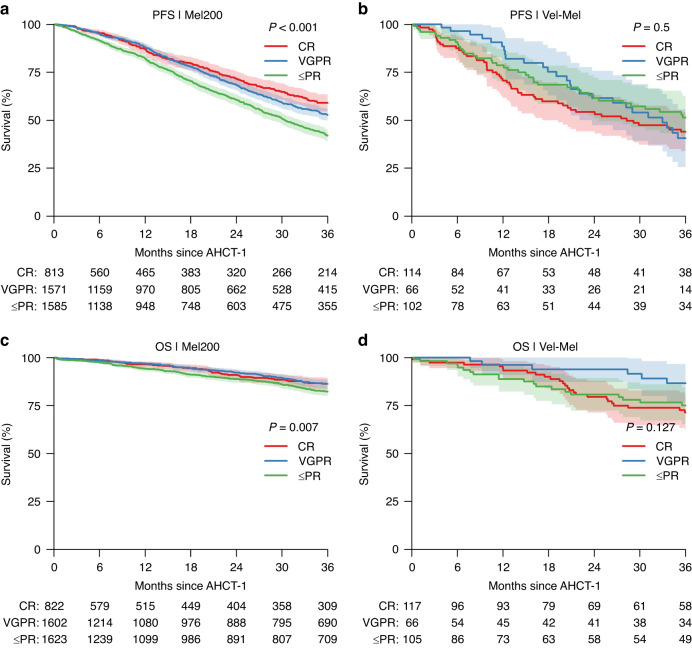
Kaplan Meier curves of PFS and OS stratified according to disease status (depth of response). Kaplan-Meier curves of (**a**) progression free survival (PFS) in patients receiving Melphalan (Mel200) conditioning, (**b**) PFS in patients receiving Velcade + Melphalan (Vel-Mel) conditioning, (**c**) overall survival (OS) in patients receiving Mel200 conditioning and (**d**) OS in patients receiving Vel-Mel, stratified by disease status at transplant (AHCT-1). Disease status is categorized as complete remission (CR), very good patial remission (VGPR) and partial remission (PR) or less. Corresponding log-rank p-values are indicated in the plots. Survival probabilities are represented as percentages, with the 95% confidence intervals indicated as shaded regions. The corresponding log-rank p-value is indicated in the plot. Below the time axis are the number of patients at risk at indicated timepoints, in each group.

As shown in Table 2, patients achieving CR/VGPR before the start of conditioning have a superior 3-year PFS when compared to those achieving PR or less (54% (51–56%) and 43% (40–46%) respectively, p < 0.001) and also have a superior 3-year OS (86% (84–87%) and 82% (80–84%) respectively, p = 0.002). Figure 2 illustrates the effect of depth of response on PFS and OS based on the conditioning regimen. After Mel200, the 3-year PFS is significantly better in those who achieved a CR when compared to VGPR and <=PR (59% (55–63%), 53% (50–56%) and 42% (39–45%) respectively, p < 0.001). Conversely, in patients treated with Vel-Mel, outcomes are similar in patients in CR, VGPR and <=PR (44% (34–54%), 41% (26–56%) and 51% (40–63%) respectively, p = 0.5) which may be due to sample size.

Patients treated with Vel-Mel were significantly more likely to achieve CR/VGPR post-transplant than patients treated with Mel200. This effect is observed both in patients in CR/VGPR and patients in <CR/VGPR before the transplant. The 3-year cumulative incidence of CR/VGPR after AHCT-1 (regardless of disease status before AHCT-1) was as follows: Mel200: 48% (46–50%) vs. Vel-Mel: 62% (55–69%); p < 0.001. The median time to CR/VGPR was similar in Mel200 and Vel-Mel treated patients (4.7 (4.2–5.2) months and 3.9 (3.2–4.5) months respectively). After reaching CR/VGPR, PFS and OS in patients treated with Vel-Mel and Mel200 is comparable.

**Fig. 3:**
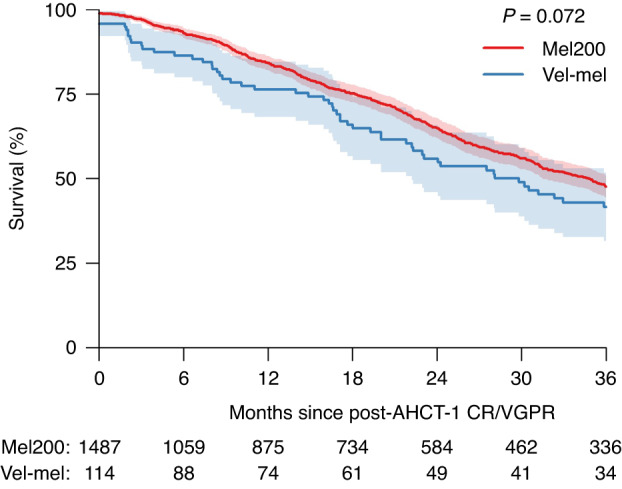
Kaplan-Meier curve of progression free survival after a best response of complete remission (CR) or very good partial remission (VGPR) after transplant (AHCT-1). Survival probabilities are represented as percentages, with the 95% confidence intervals indicated as shaded regions. The corresponding log-rank p-value is indicated in the plot. Below the time axis are the number of patients at risk at indicated timepoints, in each group.

There was a trend to a superior 3-year PFS in Mel200 patients who had achieved a CR/VGPR post-AHCT (Mel200 48% (44–51%) vs. Vel-Mel 42% (31–52%); p = 0.07; Fig. 3). 3-year OS does not differ significantly between those who achieve CR/VGPR post-AHCT (Mel200: 86% (84–88%) vs. 81% (73–89%); p = 0.17). The 3-year PFS and OS in those who achieved PR post-AHCT were similar in the Mel200 and Vel-Mel groups (PFS: 35% (32–38%) and 37% (20–54%), p = 0.8 and OS: 80% (77–83%) and 72% (57–87%), p = 0.4). Patients treated with Vel-Mel were less likely to receive a subsequent transplant without a relapse in between (12/193 in Vel-Mel and 244/1772 in Mel200 respectively). Patients treated with Vel-Mel were equally likely to relapse within 3 years after having reached a CR/VGPR (3-year relapse incidence in Mel200 and Vel-Mel: 51% (47–54%) and 55% (45–66%) respectively, p = 0.15.), and were equally likely to relapse after only having achieved a PR (63% (60–66%) and 61% (43–78%) respectively, p = 0.7). In the high-risk cytogenetics group (n = 314), the rates of CR/VGPR were similar in the Vel-Mel and Mel200 cohorts prior to AHCT-1 (71.4 and 70.1%, p = 0.99), and at Day +100 post AHCT-1 (52.9% and 63.7%, p = 0.53). The three-year PFS in this subgroup was also similar (38% (14–62%) in Vel-Mel and 39% (32–46%) in Mel200, p = 0.7) (Supplementary Fig. 1), suggesting that this intensified conditioning regimen did not improve the PFS in patients with high-risk cytogenetics.

## Discussion

High dose melphalan followed by AHCT remains the standard of care for younger more fit patients with NDMM despite the recent introduction of highly potent induction regimens. Many groups have attempted to further reduce the incidence of post-transplant relapse by intensifying the conditioning regimen with additional novel chemotherapeutic agents.

This EBMT registry-based analysis assessed the impact of the addition of bortezomib to standard Mel200 conditioning during a first AHCT, thereby evaluating ‘real world’ outcomes. Analysis of baseline characteristics revealed that patients selected for Vel-Mel were more fit, younger, were less likely to have an IgG isotype and had received similar rates of bortezomib-based induction regimens when compared with the Mel200 group. Furthermore, Vel-Mel patients achieved deeper responses than the Mel200 patients, both post-induction prior to AHCT (CR: 40.6% vs. 20.3 % and VGPR: 22.9 % vs. 39.6% respectively, p < 0.001) and by day 100 post-AHCT (CR/VGPR: 70.2% vs. 57.7%, p < 0.001), raising the possibility that they had more proliferative and thereby responsive disease but also that Vel-Mel may have been a factor in their superior post-AHCT response. However, intensification of melphalan with bortezomib did not significantly improve either PFS or OS, irrespective of baseline characteristics. Of note, fewer Vel-Mel conditioned patients proceed to a second AHCT when compared to the Mel200 group. In addition, despite similar 3-year PFS in both groups, OS was inferior in Vel-Mel recipients when compared to Mel200 patients. This may be partially attributable to more frequent second AHCTs in the Mel-200 patients.

In this retrospective analysis, where data on the duration of induction, maintenance, FISH, or ISS were incomplete in at least 50% of patients, it is not possible to definitively attribute any differences in PFS or OS to the conditioning alone. However, the response status prior to AHCT-1 and the Day +100 responses show that Vel-Mel patients were more fit, younger and more drug-sensitive, achieving a similar PFS despite a lower likelihood of having proceeded to a second AHCT-2. The superior post-AHCT response was not maintained in the Vel-Mel treated patients which may be attributable to a number of factors including biologically high-risk disease or differing post-AHCT consolidation and maintenance approaches. However, maintenance treatment was not uniformly available in EBMT-affiliated centres throughout the study period and this heterogenous availability of maintenance strategies complicates interpretation of the data. However, since patients are included from sites who treat patients with either Mel200 or Vel-Mel, it is unlikely that patient chosen to receive intensified Vel-Mel for AHCT-1 conditioning would then have had less intensive post-transplant treatment approaches, the poorer outcomes in this group more likely reflects progression of a biologically more aggressive disease.

To ascertain the role of Vel-Mel in patients with cytogenetically high-risk disease, a separate sub-analysis was performed in 314 high-risk patients. This did not detect any benefit from the addition of bortezomib to standard Mel200 conditioning. The median PFS was 29.2 months (23.1-34.7 months) with Mel200 as opposed to 21.9 months (12.2 –undefined) in the Vel-Mel group. This result can be compared with the subset analysis from the PETHEMA trial where Busulfan-Melphalan (Bu-Mel) was compared with Mel200 [19]. High-risk cytogenetics including del(17p), t(4;14), t(14;16), t(14;20), 1q amp, and del(13q) were found in 62 patients (Bu-Mel n = 32; Mel200 n = 30). Median PFS was 44.7 months in the Bu-Mel group compared to 25.7 months in the Mel200 group (p = 0.044), hence showing similar Mel200 PFS results in both this EBMT analysis and the PETHEMA study in cytogenetically high-risk patients.

The Arkansas group also investigated the role of bortezomib in patients with high-risk MM, combining bortezomib and thalidomide in the Total Therapy-4 Light protocol. Patients presenting with a GEP-51 high-risk profile achieved superior PFS and OS rates when compared to historical TT-3 protocols, an effect which was not achieved with the standard TT-4 protocol which lacked Bortezomib/Thalidomide in conditioning [17, 22]. In the EBMT study by Auner et al., it was found that higher dose Mel 200 (vs Mel140) appeared to be required when there was an inferior response to induction [4]. In this study, we observed both higher pre-AHCT and post-AHCT response rates in Vel-Mel patients. In the univariate analysis, we found a lower risk of relapse in Vel-Mel when compared to Mel200 patients if the response at AHCT was CR. Furthermore, pre and post-AHCT depth of responses were also better among patients conditioned with Vel-Mel. Although an inferior depth of pre-AHCT or post-AHCT response was associated with an increased relapse rate in all patients regardless of conditioning this discrepancy within Vel-Mel patients may be attributable to either the clinical features that led to selection of Vel-Mel, not identifiable in the current study, or due to less frequent AHCT-2 after Vel-Mel. To ascertain the role of this possibility further, a MVA integrating AHCT-2 as a competing event was performed. The results were almost identical with the initial MVA highlighted above, excluding the influencing role of AHCT-2. In addition, in the MVA, Vel-Mel, cytogenetic high-risk and lack of CR post-induction were associated with shorter PFS. It therefore appears that intensification may be beneficial in a sub-category of patients who are sensitive to bortezomib. This result is in accordance with earlier in vitro studies demonstrating a strong synergism between melphalan and bortezomib [13].

The IFM group performed a retrospective study analysing Vel-Mel conditioning in comparison with historical controls [15]. Bortezomib was administered on days -6, -3 and +1, +4 at a dose of 1.0 mg/m2 intravenously. An increase in post-AHCT-1 response rates in 54 patients treated with Vel-Mel was observed (35% vs 11%; P = 0.001). Prompted by these positive findings, a phase III prospective, open label, randomized study comparing Vel-Mel (n = 154) with Mel200 (n = 146) was performed in 2015 and 2016 [21]. However, in this study with bortezomib-based induction and VDT consolidation for all patients, no advantage of the Vel-Mel regimen in terms of response, PFS or OS was reported. Outcomes were found to be similar: stringent CR/ CR rates at Day +60 post-transplant: 22.1% vs 20.5% (P = 0.844), undetectable measurable residual disease rates: 41.3% vs 39.4% (P = 0.864), median PFS: 34.0 months vs 29.6 months (adjusted HR, 0.82; 95% CI, 0.61-1.13; P = 0.244), and estimated 3-year OS: 89.5% in both arms (hazard ratio, 1.28; 95% CI, 0.62-2.64; P = 0.374). In our current study, the 18-month PFS was 66% (60-73) in Vel-Mel treated patients, compared to 75% (74-77%) in Mel200 patients. This may possibly be due to the use of more doublet-based regimens compared to 98% VCD or VDT as induction/consolidation in the IFM study that occurred in more recent years. However, regarding OS at 18 months, both studies are comparable: 89% (85-93%) versus 93% (92-94%). In our study, we observed similar PFS outcomes following Vel-Mel compared to Mel200 with fewer needing a subsequent AHCT.

Regarding high-risk cytogenetics, the median OS and PFS overall was 81.9 months (64 – undefined upper bound) and 28.6 months (22.5-33.5), respectively. By comparison, in the prospective phase III IFM study, 16.6% (Vel-Mel) and 15.3 % (Mel200) of subjects belonged to the high-risk group, respectively; however, the authors did not report outcomes for this subgroup [21]. More recently, the UK Myeloma Group published a study of 103 MM patients identified as high-risk by either (1) Gene Expression Profiling, (2) extra-medullary disease or (3) primary plasma cell leukemia (pPCL) (acronym OPTIMUM ISRCTN16847817) [23]. The aim was to improve outcomes for this high-risk MM group. All patients received Vel-Mel conditioning and continuous consolidation with bortezomib-containing RVd regimen following induction with Daratumumab-VRCd. This non-randomized study results demonstrated very high response rates at both pre- and post-AHCT time points and PFS improvement at 18 months over 117 matched historical comparator patients from the UK Myeloma XI study.

In conclusion, this large retrospective EBMT registry-based study compared the outcomes of 292 Vel-Mel patients with 4,092 Mel200 patients. Although there was an improvement in the depth of response post-transplant, Vel-Mel intensified conditioning was not associated with superior PFS or OS. Based on this real world registry data, it appears that Vel-Mel intensification may have been preferentially selected for use in younger, more fit patients. Higher risk MM is also likely to have been a key factor in this choice. These differences in baseline population characteristics may have abrogated any possible effect of the more intensified conditioning on PFS or OS. Moreover, in the absence of an adequately sized prospective randomized study that includes sufficient numbers of high-risk MM patients, it is not possible to rule out a potential positive benefit from the intensification of conditioning with novel agents. In summary, based on our findings as well as the IFM prospective study, there is no evidence to support the routine use of Vel-Mel conditioning.

### Supplementary information

supplementary figure 1

## Data Availability

Study related additional unanimous data may be shared upon request in future.
