# Supplementary material for: In the era of Bortezomib-based Induction, intensification of Melphalan-based conditioning with Bortezomib does not improve Survival Outcomes in newly diagnosed Multiple Myeloma: a study from the Chronic Malignancies Working Party of the EBMT
Source: Bone Marrow Transplant. 2024 Jan 31;59(4):526–33. doi: 10.1038/s41409-023-02160-8 (PMC10994834; doi:10.1038/s41409-023-02160-8)

Supplementary figure 1 Kaplan-Meier curve of progression free survival within patients with high risk cytogenetics, stratified by Melphalan (Mel200) and Vel-Mel (Velcade + Melphalan) conditioning at first autologous hematopoeitic stem cell transplantation (AHCT-1). Survival probabilities are represented as percentages, with the 95% confidence intervals indicated as shaded regions. The corresponding log-rank p-value is indicated in the plot. Below the time axis are the number of patients at risk at indicated timepoints, in each group.


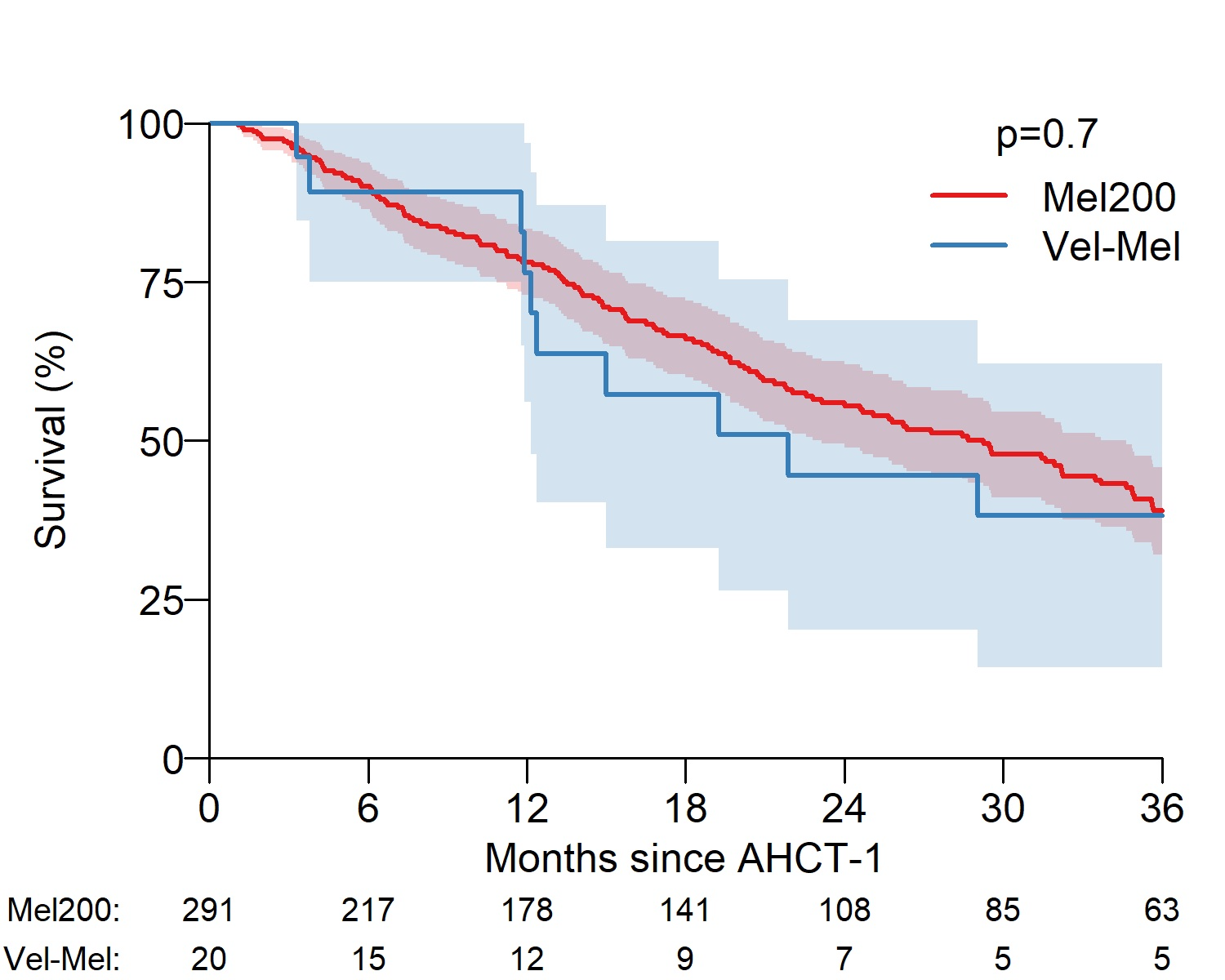

Supplement: Supplementary file 1 — supplementary figure 1 [file 41409_2023_2160_MOESM1_ESM.docx]
